# Oxidative Stability of Side-Streams from Cod Filleting—Effect of Antioxidant Dipping and Low-Temperature Storage

**DOI:** 10.3390/md21110591

**Published:** 2023-11-13

**Authors:** Ann-Dorit Moltke Sørensen, Haizhou Wu, Grethe Hyldig, Niels Bøknæs, Ole Mejlholm, Ingrid Undeland, Charlotte Jacobsen

**Affiliations:** 1 National Food Institute, Technical University of Denmark, 2800 Kongens Lyngby, Denmarkchja@food.dtu.dk (C.J.); 2 Food and Nutrition Science, Department of Life Sciences (LIFE), Chalmers University of Technology, 41296 Gothenburg, Sweden; 3 Royal Greenland, 9230 Svenstrup, Denmark

**Keywords:** fish, by-products, rest raw material, Duralox MANC-213, rosemary extract, valorization, rancidity

## Abstract

Currently, side-streams (e.g., head, backbone, tail, and intestines) generated in the fish processing industry often end up as low-value products for feed applications or even as waste. In order to upcycle such side-streams, they need to be preserved to avoid oxidative degradation of the lipids between the generation point and the valorization plant. In the cod filleting industry, three main solid side-streams: viscera, heads, and backbones, are obtained. Hence, this study aimed to identify the most efficient antioxidant for preserving the cod side-streams using a dipping-based strategy prior to pre-valorization storage at low temperatures (ice and frozen storage). The dipping solutions evaluated contained: (i) a lipophilic rosemary extract (0.05% and 0.2% in 0.9% NaCl), (ii) Duralox MANC (a mixture of rosemary extract, ascorbic acid, tocopherols, and citric acid; 2% in 0.9% NaCl), and (iii) NaCl (0.9%) *w*/*w* solution. One group was not dipped. No dipping and dipping in NaCl were included as controls. The results showed a positive effect of dipping with solutions containing antioxidants as measured by peroxide value (PV), TBA-reactive substances (TBARS), and sensory profiling, e.g., rancid odor. Moreover, the oxidative stability increased with decreased storage temperature. The cod side-streams were in general most efficiently preserved by Duralox MANC, followed by the lipophilic rosemary extract (0.2%), compared to no dipping and dipping in NaCl solution and the lower concentration of the lipophilic rosemary extract (0.05%). The efficiency of the antioxidant treatments was independent of the side-stream fraction and storage temperature. Thus, using antioxidant dipping combined with low temperature storage is an efficient preservation method for maintaining the quality of the lipids in cod solid side-streams during their pre-valorization storage.

## 1. Introduction

Global fish production was estimated to be 179 million tons in 2018, whereof 156 million tons were used for direct human consumption [1]. However, the amount that actually ends up at the consumer is considerably lower since significant amounts of by-products (also referred to as side-streams, i.e., head, backbone etc.) are generated through fish processing. Thus, ca. 50–70% of the fish weight ends up as side-streams. These side-streams are used for low-value products, such as fish meal, or may even be discarded into the sea. However, they contain valuable compounds, which could potentially be used for human consumption.

A continuous increase in the global population calls for an increased utilization degree of current resources to improve the sustainability of fisheries and aquaculture. Many researchers have evaluated the potential to valorize fish side-streams for the production of healthy lipids [2,3] and for the production of gel-forming proteins via the pH-shift-process [4,5] as well as for the production of hydrolyzed proteins [6,7,8], including hydrolysates with different functional properties, such as antioxidant, emulsifying, and antimicrobial properties [9,10,11]. However, to obtain a high-quality end product that can be used for human consumption, it is crucial to maintain the quality of the side-streams until further processing. This is especially true where logistic challenges may occur, e.g., if the side-streams will be further processed at other locations than that at which they were generated [12]. Fish lipids are highly susceptible to oxidative deterioration due to the content of long chain polyunsaturated fatty acids (LC PUFAs) such as eicosapentaenoic acid (EPA) and docosahexaenoic acid (DHA) together with highly pro-oxidative heme-proteins. Oxidative deterioration will cause rancidity of the side-streams, which will limit their potential utilization within the food chain. Thus, efficient methods for preserving fish side-streams are crucial for further valorization. Only a few studies have been carried out regarding pre-processing preservation of fish side-streams using techniques such as decreased storage temperature, antioxidant treatment [7,13,14,15], and acidification [12]. When dipping the side-streams into antioxidant solution, the surface blood of the material will be washed away [13], thereby decreasing the degree of hemoglobin (Hb)-mediated lipid oxidation. Additionally, a thin layer of antioxidant will cover the surface at which the oxidative attack normally occurs. Dipping of herring (*Clupea harengus*) side-streams in 2 or 5 ratios of antioxidant solution previously revealed efficient preservation ability even when the solution was re-used up to 10 times [14,16].

In Greenland, large volumes of fresh cod *(Gadus morhua*) side-streams are generated. Due to logistics challenges such as lack of infrastructure and long transport distances between producers of side-streams and potential users of the side-streams, the quality of the fresh side-streams must be maintained for an extended period of time. The aim of this study was to evaluate the oxidative stability of cod solid side-streams during storage at different temperatures when combined with different antioxidant treatments. The antioxidants evaluated were commercially available blends based on rosemary extracts. One of the blends, however—Duralox MANC—also included other antioxidants (ascorbic acid, tocopherols, and citric acid), which allowed evaluation of the combined antioxidant effect. The concentrations of the blends were selected based on previous work to stabilize herring side-streams [14,16]. Additionally, a control treatment without antioxidant, i.e., dipping in NaCl and another control with no dipping, were included to evaluate the effect of dipping and antioxidant in the dipping solution. The efficiency of the dipping treatments was evaluated both during ice and frozen storage based on the development of primary and secondary oxidation products measured as peroxide value (PV) and TBA-reactive substances (TBARS), respectively. In addition, sensory evaluation was applied to evaluate the efficiency and suitability of the antioxidant blends.

## 2. Results

### 2.1. Crude Composition: Dry Matter, Protein and Oil Contents of Cod Solid Side-Streams

The compositions of the three different side-streams (backbone, head, and viscera) are summarized in Table 1. Results revealed significant variation in the composition of the side-stream fractions. The variation in dry matter and oil was greater for the viscera fraction (larger standard deviations) than for backbone and head, which may be due to the inherent inhomogeneity of the former fraction, as it consists of intestines, liver, stomach, roe, etc., with varying sizes and amounts. The dry matter and oil contents ranged between 23–35% and 1–23%, respectively, in the different side-streams, with significantly higher levels of both in the viscera compared to the head and backbone. Protein contents (10–15%) differed significantly between all the three side-stream fractions, with the lowest content in the viscera and highest content in the backbone fraction.

The relative contents of the n-3 LC PUFAs—EPA and DHA—were 9–15% and 11–21%, respectively. This means that the content of EPA and DHA together accounted for 21–36% of total fatty acids measured. The proportions of EPA and DHA were significantly higher in the frame fraction, which was the fraction with the lowest oil content. If the side-streams are handled improperly, these n-3 LC PUFAs will oxidize, which will result in decreased quality of the side-streams.

After antioxidant-dipping, the three different side-stream fractions had similar amounts of dry matter and protein, as observed for the initially characterized side-streams. In contrast, the oil contents of the head and viscera were slightly lower after dipping than before dipping (Table S1 vs. Table 1). This is suggested to be ascribed to variations in the side-stream due to biology, season, size, etc.

### 2.2. Initial Hb Content in the Different Cod Side-Streams

**Figure 1:**
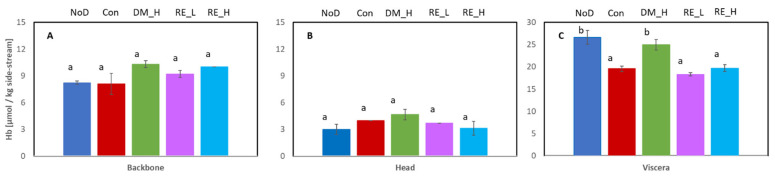
Hemoglobin (Hb) (µmol Hb/kg side-stream) in the three side-streams with the different treatments; (**A**) backbone, (**B**) head, and (**C**) viscera. NoD: no dipping; Con: dipped in 0.9% NaCl solution; DM_H: dipped in 2% Duralox MANC dissolved in 0.9% NaCl solution; RE_L: dipped in 0.05% rosemary extract dissolved in 0.9% NaCl solution; RE_H: dipped in 0.2% rosemary extract dissolved in 0.9% NaCl solution. Different letter for a side-stream indicates significant differences (*n* = 2).

Hb-bound iron is known to be a crucial parameter for the oxidative stability of the side-streams [15]. Therefore, the content of Hb in the three different side-stream fractions subjected to different treatments was measured. Hb-derived iron is not only able to catalyze the initiation of lipid oxidation, but it also takes part in the decomposition of pre-formed lipid hydroperoxides into volatile secondary oxidation products that give rise to off flavors. The Hb content was significantly higher in the viscera fractions compared to the head and backbone fractions (Figure 1).

For the backbone and head, the Hb content ranged between 8.2–10.3 µmol/kg and 3.0–4.6 µmol/kg, respectively. There were no significant differences between the treatments (Figure 1A,B). Regarding the viscera fraction, the Hb content after the different treatments ranged from 18.3–26.6 µmol/kg, with significantly higher contents in the fraction without dipping (NoD) and in the fraction dipped in Duralox MANC (DM, Figure 1C).

### 2.3. Effect of the Antioxidant Dipping on Lipid Oxidation

The lipid content was different in the different side-stream fractions and since the quality of the lipid fraction per se was the focus of this study, all the results for PV, tocopherols, and TBARS were calculated relative to the oil content instead of relative to the sample weight. Free fatty acids (FFA) are generated from lipase activity, which cleaves the triglycerides (TG) to FFA and mono- and di-glycerides as well as from phospholipase activity cleaving the phospholipids (PL) to FFA and lyso-PL. When derived from TG, FFA oxidizes more easily in the free form, while the opposite has been seen regarding the hydrolysis of PL [17,18]. Since no heat treatment has been used to inactivate endogenous enzymes, FFA may be generated in the cod side-streams during storage.

#### 2.3.1. Backbone

**Figure 2:**
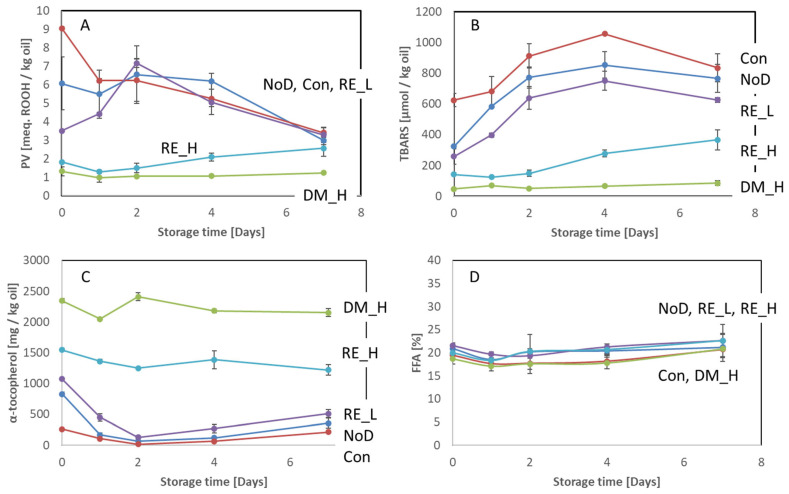
Antioxidant treated and non-treated cod backbones stored at 5 °C on ice for 7 days. (**A**) PV, (**B**) TBARS, (**C**) α-tocopherol, and (**D**) FFA. NoD: no dipping; Con: dipped in 0.9% NaCl solution; DM_H: dipped in 2% Duralox MANC dissolved in 0.9% NaCl solution; RE_L: dipped in 0.05% rosemary extract dissolved in 0.9% NaCl solution; RE_H: dipped in 0.2% rosemary extract dissolved in 0.9% NaCl solution. The results are expressed on an oil basis and show average ± standard deviation (*n* = 2).

The changes in PV, TBARS, α-tocopherol, and FFA during ice storage of antioxidant-treated cod backbone are shown in Figure 2.

The initial level of peroxides in cod backbones was different for the different treatments (Figure 2A). At time 0, significantly higher PV was observed for the control and no dipping than for 0.2% rosemary extract (RE) and 2% DM, which had the lowest initial PVs. Depending on the antioxidant treatment, different patterns for the development of PV during storage were observed. The PV significantly decreased in the control during storage from 9.0 to 3.4 meq. ROOH/kg oil. No dipping and 0.05% RE had a slight increase (day 0 to 2) in PV with time, whereafter it decreased (Day 2 to 7); changes were significant for 0.05% RE. However, for untreated backbone, storage time had no significant effect. For 0.2% RE, the PV increased slightly during storage, and at Day 7, it was significantly higher than Day 0. Cod backbone treated with 0.2% RE had significantly lower PV than backbone treated with 0.05% RE both initially and during ice storage. However, at Day 7, the PV level was not significantly different from the other treatments, except for the backbone treated with 2% DM. There was no significant difference in the PV level for backbone treated with 2% DM through the 7 days of ice storage.

For the TBARS, the initial concentrations on an oil basis differed significantly between the treatments applied (Figure 2B). Similarly as observed for the PV, DM (2%) had a significantly lower initial concentration of TBARS than 0.05% RE, no dipping, and control, while 0.2% RE had a significantly lower initial concentration of TBARS than no dipping and control. The concentration of TBARS increased with storage time, with the increment being dependent upon the antioxidant treatment. Only a minor increment in TBARS was observed for 2% DM (47–85 µmol/kg oil between day 0 to day 7). However, it was significant. A significant increase in TBARS was observed after four days of storage for 0.2% RE, after two days of storage for control and 0.05% RE, and after one day of storage for no dipping. After seven days of storage, the concentration of TBARS was lowest in 2% DM, followed by 0.2% RE. For 2% DM, the concentration was significantly lower throughout the storage than the other treatments, whereas for 0.2% RE, the concentration was significantly lower than for no dipping and dipping in NaCl only.

The four tocopherol homologues were measured in the different cod backbones. Only alpha tocopherols were present in the non-antioxidant treated cod backbone while 2%-DM-treated backbone also contained gamma- and delta-tocopherols. Gamma-tocopherols in DM decreased from 2696 mg to 2538 mg/kg oil (not significant), whereas the concentration of delta-tocopherol was stable during storage (724–726 mg/kg oil). The initial alpha-tocopherol concentrations were influenced by the treatments (Figure 2C), where DM, 0.2% RE, and 0.05% RE had higher concentrations due to tocopherol content in the antioxidant extracts. During storage, the concentration decreased significantly for all treatments. At the end of the storage, the concentration was significantly higher in DM followed by 0.2% and 0.05% RE and the lowest concentration was found in non-antioxidant treated backbones with insignificant differences between the two latter and between 0.05% RE and no dipping.

The FFA measured in backbones for the different treatments is shown in Figure 2D. There were small differences, but these were not significant between treatments and storage points within treatments.

When the cod backbones treated with different antioxidants were stored frozen (−20 °C), similar effects of the different treatments were observed as during ice storage. The most efficient treatment in terms of maintaining the quality was again DM followed by 0.2% RE (Supplementary Figure S1). DM inhibited the development of PV and TBARS for 3 months, whereas all other treatments gave rise to a significant increase from 0 to 3 months of storage. Opposite to the findings during ice storage, a significant increase was observed for the level of FFA, however, the initial level and levels during storage did not differ significantly between treatments. The differences in the initial FFA and the FFA-development during storage could be due to the freeze–thawing taking place prior to the ice storage experiment. It has also been reported that, e.g., phospholipases have a maximum activity at −4 °C [19], and thus, FFA formation can even be accelerated during freezing/frozen storage [20]. Reasons for this can be the cellular decompartmentation resulting from freezing/freeze–thawing as well as up-concentration of reactants in the unfrozen pool of water [21].

#### 2.3.2. Head

**Figure 3:**
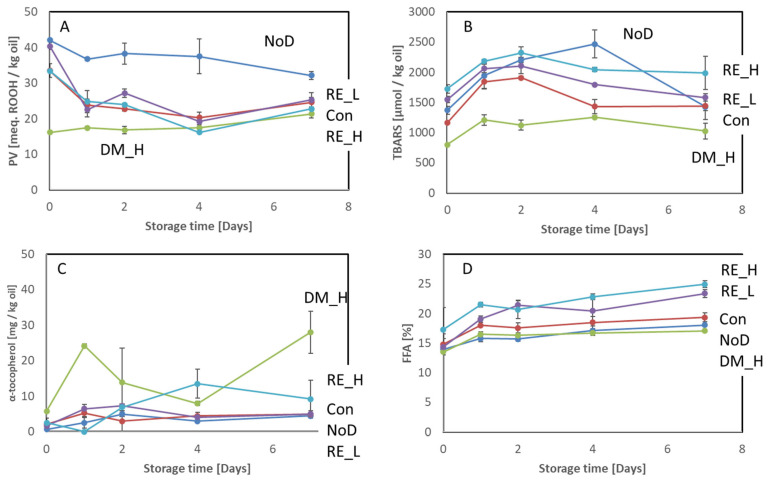
Antioxidant-treated and non-treated cod heads stored at 5 °C on ice for 7 days. (**A**) PV, (**B**) TBARS, (**C**) α-tocopherol, and (**D**) FFA. NoD: do dipping; Con: dipped in 0.9% NaCl solution; DM_H: dipped in 2% Duralox MANC dissolved in 0.9% NaCl solution; RE_L: dipped in 0.05% rosemary extract dissolved in 0.9% NaCl solution; RE_H: dipped in 0.2% rosemary extract dissolved in 0.9% NaCl solution. The results are expressed on an oil basis and show average ± standard deviation (*n* = 2).

The developments of PV, TBARS, α-tocopherol, and FFA during ice storage of antioxidant treated and non-treated cod heads are shown in Figure 3.

The initial PV was significantly lower for DM-treated cod head compared to the other treatments (Figure 3A), but PV increased significantly between Days 4 and 7. The other treatments resulted in a significant decrease in PV during storage except for the non-dipped sample, which showed no significant changes in PV during storage. The levels of TBARS were significantly higher on Day 2 than the initial levels for all the cod heads independent of treatment (Figure 3B). Thereafter, the concentration decreased except for the non-dipped sample; here a decrease in TBARS was observed first after Day 4. DM-treated heads had a significantly lower concentration of TBARS until Day 4. Thereafter, the TBARS level was not significantly different from the control (Days 4 and 7) and non-dipped (Day 7) heads.

Cod head had a very low amount of tocopherols, which also included the samples treated with antioxidant blends containing tocopherols. The concentration of alpha-tocopherol detected in the cod heads during storage is shown in Figure 3C. The initial concentrations for all treatments were ≤2.5 mg/kg oil, except for DM, which had a significantly higher initial concentration (5.8 mg/kg oil). Alpha-tocopherol levels in heads treated with 0.2% RE increased during storage, whereas they both increased and decreased in DM-treated heads. However, the concentrations in the head fractions were very low and the samples had different oil contents which is assumed to be the main reason for the observations. Gamma- and delta-tocopherols were only detected in cod heads treated with DM in concentrations of 27.6–31.8 and 14.0–16.7 mg/kg oil, respectively.

The FFA levels measured in heads subjected to different treatments are shown in Figure 3D. No initial significant differences between treatments were observed. The concentration of FFA increased significantly in all heads independent of treatments. At the end of the storage, DM- and non-antioxidant-treated heads had significantly lower concentrations than heads treated with RE.

Frozen storage (−20 °C) of cod heads produced similar results for the different treatments as ice storage did. The most efficient treatment in terms of maintaining the quality was DM, followed by 0.2% RE (Supplementary Figure S2). Non-dipped heads showed a high increase in PV from 10–35 meq. ROOH/kg oil, which was not observed during ice storage. Moreover, in frozen storage, the concentration of TBARS decreased during storage as opposed to ice storage. Furthermore, the concentration of FFA increased throughout the entire frozen storage, whereas in ice storage, only an increase in the initial part of the storage was observed. Similar to the findings for cod backbone, the differences in the development of PV, TBARS, and FFA during storage could be due to the additional freeze–thawing applied prior to the ice storage experiment but also to the activity of lipases during frozen storage [20,21].

#### 2.3.3. Viscera

**Figure 4:**
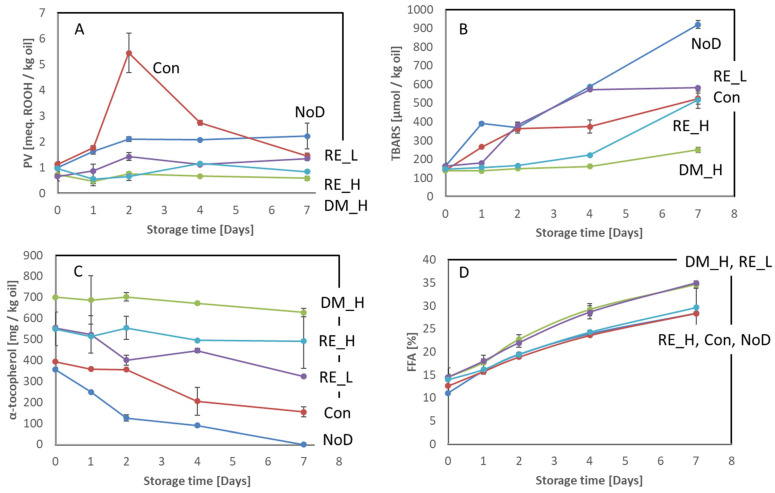
Antioxidant-treated and non-treated cod viscera stored at 5 °C on ice for 7 days. (**A**) PV, (**B**) TBARS, (**C**) α-tocopherol, and (**D**) FFA. NoD: no dipping; Con: dipped in 0.9% NaCl solution; DM_H: dipped in 2% Duralox MANC dissolved in 0.9% NaCl solution; RE_L: dipped in 0.05% rosemary extract dissolved in 0.9% NaCl solution; RE_H: dipped in 0.2% rosemary extract dissolved in 0.9% NaCl solution. The results are expressed on an oil basis and show average ± standard deviation (*n* = 2).

The development of PV, TBARS, α-tocopherol, and FFA during ice storage of antioxidant-treated cod viscera is shown in Figure 4.

The initial PV level was generally low in the cod viscera fractions, and there were no significant differences between the treatments (Figure 4A). The control dipped in NaCl showed a significant increase in PV followed by a significant decrease. The other treatments, except DM, resulted in a lower increment than the control but still significant. DM-treated viscera showed no changes in PV throughout the storage. Hence, DM treatment was the most efficient for inhibiting the development of PV followed by RE at 0.2 and 0.05%. Like PV, DM-treated viscera had the lowest TBARS concentration among the treated viscera fractions throughout the storage (Figure 4B). A significantly higher concentration of TBARS in DM-treated viscera was, however, observed at Day 7 compared to Day 0. A significantly higher concentration of TBARS was also observed for 0.2% RE-treated viscera. For non-antioxidant treated viscera, the increment of TBARS was significant already at Day 1 and for 0.05%-RE-treated viscera at Day 2.

As shown in (Figure 4C), the concentration of alpha-tocopherol generally decreased during storage. For DM and 0.2% RE, the decrease was, however, insignificant. At the end of the storage, all alpha-tocopherol was consumed in non-dipped viscera. The control dipped in NaCl had the second lowest concentration of alpha-tocopherol at the end of the storage. Hence, the antioxidant treatments reduced or inhibited the consumption of alpha-tocopherol. Similarly to the findings for backbone and head fractions, gamma- and delta-tocopherols were present in viscera fractions treated with DM (274–297 and 75–81 mg/kg oil, respectively), with insignificant changes during storage.

The concentration of FFA in ice-stored viscera fractions is shown in Figure 4D. The initial concentrations were not different among the different treatments. However, the concentration then increased at different rates in viscera fractions treated differently. At Day 4, 0.05% RE- and DM-treated viscera fractions had significantly higher FFA concentrations than the other treatments, but at Day 7, the differences were insignificant. The level of FFA generally appeared to be independent of the antioxidant applied and rather was a result of biological variations.

The concentrations of PV, TBARS, alpha-tocopherol, and FFA in viscera during frozen storage (Figure S3) showed similar results to those stored on ice. However, the differences in efficiency between DM and RE was higher during frozen storage than during ice storage based on PV and TBARS results.

### 2.4. Sensory Evaluation of the Antioxidant Preserved Cod Side-Streams

The sensory evaluation was only performed on selected treatments and storage conditions for the three different cod side-streams—backbone, head, and viscera.

**Figure 5:**
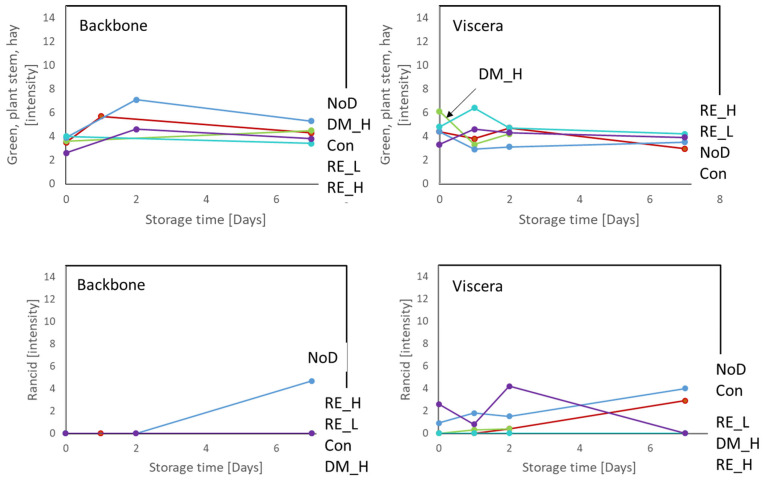
Sensory intensity of green/plant stem/hay and rancid odor for antioxidant-treated and non-treated cod backbone and viscera stored at 5 °C on ice for 7 days. NoD: no dipping; Con: dipped in 0.9% NaCl solution; DM_H: dipped in 2% Duralox MANC dissolved in 0.9% NaCl solution; RE_L: dipped in 0.05% rosemary extract dissolved in 0.9% NaCl solution; RE_H: dipped in 0.2% rosemary extract dissolved in 0.9% NaCl solution.

In the ice storage experiment, almost all sampling points were evaluated for the viscera samples, whereas samples from Day 0, Day 2 (Con, NoD, RE_H) and Day 7 were evaluated for the head and backbone. Rancid odor was detected early in the viscera samples, especially in the non-dipped viscera and viscera dipped in 0.9% NaCl solution. There was not detected rancidity in the head samples, and for the backbone samples, it was only the non-dipped backbone that reached similar intensity at the end of storage as viscera samples. The development of rancid odor was, however, prevented by DM and RE for backbone (Figure 5) and head, respectively. The positive sensory attribute “green/plant/steam/hay” increased during the first two days of ice storage, except for the samples dipped in DM and 0.2% RE (Figure 5).

During frozen storage, the sampling points at 0, 90, and 180 days were evaluated for all treatments, as was Day 140 for the non-dipped sample (Figure S4). It was seen that only 0.2% RE prevented the development of rancid odor in the viscera samples. For the backbone fractions, there was no or barely detectable rancidity for all treatments. Moreover, off odor was detected using the sensory panel for both ice- and frozen-stored side-streams. For the ice-stored non-dipped viscera, the description of off-odor was “fermented/ensilage”, whereas the description for the backbones treated with RE (0.05 and 0.2%) was “green tea/flowerish”. During frozen storage, there was no detection of “green tea”, but an “herbal tea” odor was detected for the antioxidant treated samples. The intensity of off odor during frozen storage was most pronounced in the backbone and viscera samples (Figure S4).

## 3. Discussion

The dipping technology did not reduce the level of Hb in backbone and head samples (Figure 1). The lower Hb content of viscera subjected to some of the dipping treatments may indicate that some Hb is removed by the dipping process. However, the differences could also reflect different proportions of, e.g., liver in the viscera among batches. Furthermore, the much lower content of Hb in backbone and head fractions may be caused by a higher degree of processing/rinsing of these fractions, resulting in less blood (e.g., heads were soaked in water before freezing) than in the viscera fraction. The general level of Hb in the different fractions of cod side-streams (3–27 µmol/kg) was considerably lower than the level in different fractions of herring side-streams with or without rinsing (33–70 µmol/kg) [13] and slightly lower than other measures on cod backbone (approx. 15–25 µmol/kg) [22]. This is likely related to the bleeding applied to the cod, which is not applied to small pelagic fish like herring.

Overall, a positive effect of the antioxidant treatment on the oxidative stability of the cod side-streams was observed. The DM solution was the most efficient, followed by the solution made from the lipophilic RE in high concentrations (0.2%). The RE-treated samples (0.05 and 0.2%) developed an off odor described as “green tea/flowerish”, which can limit its utilization. However, decreasing the concentration in order to reduce these notes may not result in the same degree of stabilization of the side-streams. On the other hand, the notes may not affect the final processed product, but is something that should be evaluated for each application recipe before further conclusions can be drawn.

Besides the protective effect during storage until further processing, the antioxidant may also stabilize the lipids under the actual processing of the side-streams if the antioxidant is not depleted during storage. Antioxidant treatment of herring side-streams before recovery of proteins using mechanical separation or before ensilaging had a positive effect on the stability during ice or ambient storage, respectively [23,24]. Whether pre-processing stabilization can also prevent oxidation during other types of valorization processes remains to be shown.

### 3.1. Effect of the Antioxidant Preservation Measured on Oxidative Deterioration

It is common that the efficacy of antioxidants is affected by the matrix [25]. The cod side-streams comprised different matrices. However, all antioxidant treatments prevented their oxidation and showed the same internal ranking order independent of the matrix. Even though antioxidant dipping and glazing are common preservation methods for refined fish materials, such as fillets [26], a limited number of studies have evaluated such preservation techniques for fish side-streams. These studies have mainly explored the stabilization of herring side-streams using antioxidant incubation, rinsing, and dipping [13,14,16]. The high efficiency of DM at 2% for preservation of cod side-streams was in agreement with the results obtained, with herring side-streams using 2% and 5% DM [13,14]. Contrary to our findings, dipping in a lipophilic RE at 0.05% was as efficient as DM (0.5%) for preservation of herring side-streams [16], whereas DM was more efficient than RE in the current study with preservation of cod side-streams. Antioxidant concentrations of DM (2%) and RE (0.05 and 0.2%) applied in our study were higher. However, the difference between DM and RE is similar (10-fold) to the preservation of herring side-stream. The different results observed between herring and cod side-streams when applying lipophilic RE is assumed to be due to compositional differences between the side-streams, e.g., different concentrations of Hb. Another explanation could also be the ratio between antioxidant solution and side-streams; a larger amount of antioxidant solution was applied for preservation of the herring compared to the amount applied for cod side-streams (1:5 and 1:2 side-stream-to-antioxidant solution, respectively).

The antioxidants applied were two commercial antioxidant blends based on rosemary extracts. However, the two blends differ in composition.DM is a rosemary preparation fortified with ascorbic acid, alpha-tocopherol, and citric acid, while the lipophilic RE is based on carnosic acid and carnosol in an oil carrier with endogenous tocopherol present. Thus, the better preservation of the cod solid side-streams with DM is anticipated to be due to synergy between different hydrophilic and lipophilic antioxidant compounds in DM compared to the lipophilic RE. Synergies between rosemary extract, ascorbic acid, α-tocopherol, and citric acid have been reported before. Citric acid has, for example, shown an additive effect with rosemary extract in sunflower oil [27]. Other phenolics, such as caffeic acid, have also been shown to work in synergy with ascorbic acid and alpha-tocopherol in fish muscle [28]. Furthermore, rosemary extract and alpha-tocopherol have shown synergies in sardine oil model systems [29]. It is expected that similar synergistic effects to those observed between caffeic acid, ascorbic acid, and alpha-tocopherol can occur between carnosic acid/carnosol and tocopherol plus ascorbic acid. Moreover, a review on rosemary as a source of antioxidants reports synergistic effects of RE in combination with, e.g., citric acid or ascorbic acid [30]. In addition, the prevention of hemin loss in herring side-streams pre-dipped in DM has been suggested to be one of the main mechanisms of action behind the observed stabilization of the herring lipids [14].

In herring side-streams, Hb-mediated lipid oxidation has been shown to be crucial for the quality degradation [26]. In the current study, results for viscera showed that the NaCl-dipped control (Con) had a slower TBARS development rate and scored lower in rancid odor intensity than the non-rinsed sample (NoD). One reason may be removal of Hb from the surface, prohibiting the breakdown of peroxides into carbonyls. The high accumulation of peroxides in this sample supports this hypothesis. For the viscera samples, this was confirmed by the amount of Hb present after dipping treatment (except DM-treated) and after no dipping (NoD) (Figure 1). However, in the backbone and head samples, the initial content of Hb was more similar for the non-dipped and dipped samples. The development of PV and TBARS in these samples was also more similar (Figure 2 and Figure 3) than was the case in the viscera samples (Figure 4). The different effects from dipping on Hb in the different side-streams may be due to the location of the Hb. The viscera fraction had high blood content on the surface, which was easily removed by the dipping compared to the backbone and head, where Hb was situated in the interior. Thus, it was difficult for the dipping solution to access. For herring side-streams, it has been demonstrated that dipping only removes a limited amount of Hb, and it was concluded that a large proportion of the residual blood was situated in the interior [14]. Altogether, the treatment of cod side-streams with the antioxidant solution did not primarily reduce the Hb concentration but possibly prevented metHb formation or heme loss [14], thereby stabilizing the lipids.

### 3.2. Effect of Storage Temperature on the Oxidative Stability of the Antioxidant Treated Cod Side-Streams

During frozen storage, the concentrations of PV and TBARS were lower than during ice storage, as expected. Ranking among the antioxidant’s efficiency was, however, similar under both storage conditions. Differences in lipid oxidation at different storage temperatures have earlier been observed for different fish raw materials, including side-streams, where lower storage temperatures have generally better preserved the lipids [31,32,33].

The sensory evaluation showed development of rancid odor in viscera treated with DM in frozen storage, whereas in ice storage, both DM and RE (0.2%) inhibited the development of rancid odor. The reason for the deviation in rancid odor for DM-treated samples stored on ice vs. under frozen conditions is unclear since the chemical measurements of lipid oxidation showed a lower degree of oxidation at frozen storage. Although samples were homogenized prior to storage, the inhomogeneous nature of the viscera could have played a role despite the fact that the storage experiments (ice and frozen) were initiated from the same initial homogenized-antioxidant-treated viscera fraction. A similar observation has been reported after frozen storage of Atlantic mackerel fillets, where PV and TBARS did not correlate with sensory [34].

## 4. Materials and Methods

### 4.1. Materials

Chemicals were of analytical grade, and solvents were of HPLC grade. The antioxidants—Duralox MANC-213 (DM) and rosemary extract (RE)—were kindly provided by Kalsec (Kalamazoo, MI, USA) and purchased from Hunan Shineway Enterprise (Changsha, China), respectively.

The solid side-streams were from cod (*Gadus morhua*) caught by a pound net fishery in October 2019 in the area of Maniitsoq (west coast of Greenland). They were brought to cages at shore and slaughtered at the factory on land. After removal of viscera, bleeding and filleting the solid side-streams were frozen in a blast freezer to −20 °C within 2 h after slaughtering. For further details about collection of the side-streams, see the section below.

#### Cod Solid Side-Streams

**Figure 6:**
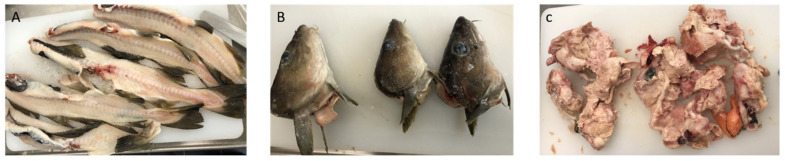
Cod solid side-streams from Royal Greenland; (**A**) backbones, (**B**) heads, and (**C**) viscera fraction after thawing (DTU).

The cod solid side-streams (backbone, head, and viscera) from the filleting process, which are normally discarded at Royal Greenland (Maniitsoq, Greenland), were collected in smaller fractions immediately after the filleting process. Backbones and viscera were collected separately in plastic bags, with approx. 1 kg/bag. The air was pressed out before closing the bags. Heads were packed one by one. Cod backbone was collected from the waste belt after the filleting machine; cod head was collected immediately after cutting and was soaked in ice water before freezing. Cod viscera was collected from the belt going to waste before it had reached the point of waste. All freshly collected side-streams were frozen after collection and shipped under freezing conditions from the factory in Greenland to Denmark. The transportation time from the sampling of the side-streams upon arrival at DTU was approx. 1 month with storage at −20 °C. At DTU, the cod side-streams were stored at ≤−40 °C until the preservation experiments. Figure 6 shows the cod side-streams after thawing at DTU.

### 4.2. Antioxidant Dipping

All dipping solutions were prepared in tap water containing 0.9% (*w*/*w*) NaCl in order to maximize the stability of red blood cells [35]. Antioxidants were added to the 0.9% NaCl solution as follows: 2% (*w*/*w*) Duralox MANC-213 (DM_H); 0.05% (*w*/*w*) rosemary extract (RE_L), and 0.2% (*w*/*w*) rosemary extract (RE_H). The rosemary extract was lipophilic, and this dipping solution was prepared with a carrier according to the method described by Wu et al. 2021 [16]. All dipping solutions were freshly prepared and stored at 4 °C for at least 5 h before use. The different dipping treatments and concentration of antioxidants applied are shown in Table 2.

The side-streams were thawed overnight in a 2 °C cold room prior to the preservation experiment. At least 6 pieces of side-stream (backbone and head) were used for each preservation treatment, and for viscera 2.1–2.2 kg (equal to two bags) was used for each preservation treatment. Thawed side-streams were dipped in antioxidant solution in the ratio 1:2 (*w*/*w*, side-streams: antioxidant solution). The side-streams were dipped for 20 s and drained in a stainless steel drainer for 1 min. After antioxidant dipping and draining, the side-streams with bones (backbone and head) were chopped using a Sakamoto kitchen knife prior to coarse homogenization using a blender (Robot Coupe, Blixer 4, Brønnum, Herlev, Denmark). The viscera fraction was homogenized directly in the blender without coarse pre-chopping.

The coarse homogenized samples were divided into smaller sample portions for longer-duration storage at −20 °C (6 months) and the portion going to ice storage was kept as short as possible at −80 °C until the experiment was initiated.

### 4.3. Storage Conditions and Sampling

For long-term storage, the samples were packed in plastic bags with 210 g in each (2–4 mm thin layers; all bags similar) and placed at −20 °C (darkness), with sampling after 0, 3, 4½, and 6 months of storage. On the sampling day, the samples were thawed (except time 0) in well-sealed plastic bags under cold running water to make the thawing relatively fast. Before sampling for different analyses, all the liquid was squeezed out from the inside of the bag. The samples were homogenized to a fine paste using liquid nitrogen and a blender. Thereafter, the samples were divided into smaller portions according to the different analyses and stored at −80 °C until analyses were performed.

Before initiating the cold storage experiment on ice, the minced side-streams were thawed. The thawing was carried out in well-sealed plastic bags under cold running water. All of the mince and liquid (leaked from the side-stream under freezing-thawing) was squeezed out from the inside of the plastic bags into a beaker. Thereafter, the samples were mixed before sampling. The mixed side-stream was divided into brown glasses (500 mL) with a 210 g sample covered with lids and placed on ice in a cold room (5 °C, darkness) for storage with regular sampling (0, 1, 2, 4, and 7 days).

On the sampling day for both ice and frozen storage, the side-streams were minced in a grinder. For the head and backbone, liquid nitrogen was used to help obtain a homogeneous paste before the sample was divided in small bags for each analysis and stored at −80 °C until further analysis. All analyses were performed in duplicate (n = 2).

Samples for sensory analysis were also stored at −80 °C until analysis could be performed. This allowed all sample points to be evaluated at the same time.

### 4.4. Dry Matter

Side-stream samples were weighed and placed in an oven (102–105 °C, 20–24 h). The dry matter content was determined gravimetrically and expressed as a percentage of the sample weight (*w*/*w*).

### 4.5. Oil Content

The oil was extracted using chloroform and methanol according to the Bligh and Dyer method [36] with a reduced amount of solvent [37]. After oil extraction and the evaporation of chloroform, the oil content was determined gravimetrically. The results are reported as a percentage of the sample weight. The obtained lipid extract was also used for analyses of PV, tocopherol content, fatty acid composition, and FFA.

### 4.6. Peroxide Value (PV)

PV in the lipid extracts was determined using a colorimetric ferric-thiocyanate method as described by Shanta and Decker 1994 [38]. Chloroform was evaporated for the lipid extracts, and the oil re-dissolved in a mixture of chloroform and methanol (7:3, *v*/*v*). The PV was measured on a spectrophotometer at 500 nm (Shimadzu UV1240, Shimadzu Scientific Instruments, Columbia, MD, USA). Results were reported as meq. peroxides (ROOH) pr. kg oil.

### 4.7. Tocopherols

Lipid extracts were evaporated to remove chloroform, re-dissolved in heptane, and analyzed for tocopherol content using an HPLC (Agilent 1100 Series, Agilent Technology, Palo Alto, CA, USA) equipped with a silica column (Waters (Dublin, Ireland), 150 mm, 4.6 mm, 3 µm silica film) according to the AOCS Official Method Ce 8-89 (1997) [39]. Tocopherol isomers were quantified according to external tocopherol standards using single-point calibration. Results were reported as mg tocopherol/kg oil.

### 4.8. Fatty Acid Methyl Esters (FAME)

Lipid extracts obtained were evaporated under nitrogen. Toluene and heptane with internal standard (C23:0) (1:3, *v*/*v*) were added, and the lipids were methylated in a one-step procedure in an acid-catalyzed process with a 20% boron trifluoride reagent. This process was accelerated using a microwave (Multiwave3000 SOLV, Anton Paar, Graz, Austria; 5 min heating at 500 W; 10 min cooling) with a 64MG5 rotor [40]. FAMEs were dissolved in heptane, and the compositions of methyl esters were analyzed on a GC (HP 5890A, Agilent Technology, Palo Alto, CA, USA) according to the AOCS Official Method Ce 1b-89 (1998) [41]. For separation, a DB-wax column (10 m × ID 0.1 mm × 0.1 µm film thickness, J&W Scientific, Folsom, CA, USA) was used, and the temperature program of the GC oven was as follows: 160–200 °C 10.6 °C/min, 200 °C kept for 0.3 min, 200–220 °C 10.6 °C/min, 220 °C kept for 1 min, 220–240 °C 10.6 °C/min and kept at 240 °C for 3.8 min. Results were reported as percentages of total fatty acids. The FAMEs were analyzed to evaluate the content of especially EPA and DHA.

### 4.9. Determination of Thiobarbituric Acid Reactive Substances (TBARS)

Fifteen g samples were weighed, and a 7.5% trichloroacetic acid (TCA) solution with 0.1% EDTA and 0.1% propyl gallate was added. The sample was then mixed using an Ultra Thurrax (approx. 1000 rpm; 15 s) until homogeneous. The homogenate was then filtered. Some of the filtered extracted was mixed with 0.02 M TBA reagent (2-thiobarbituric acid) in one glass and with water in another glass (blank) in the ratio 1:1 (*v*/*v*). Thereafter, the samples were heated at 90 °C for 40 min. After cooling of the heated samples, the absorbance was measured at 530 nm (Shimadzu UV1240, Shimadzu Scientific Instruments, Columbia, MD, USA). An external calibration curve was performed using 1,1,3,3-tetraethoxypropane (TEP, malondialdehyde bis (diethyl acetal)) dissolved in 7.5% trichloroacetic acid (TCA) as the standard to calculate the concentration of TBARS. The results were related to the oil content and thus expressed as µmol malondialdehyde/kg oil.

### 4.10. Free Fatty Acids (FFA)

Lipid extract was mixed with ethanol (25 mL), and some drops of indicator were added. Then, the sample was titrated with 0.1 M NaOH until a faint pink color appeared. The volume of NaOH used for titration was used to calculate FFA (%). The results were reported as the amount of FFA (%) as oleic acid equivalents on oil basis.

### 4.11. Hemoglobin Content

The Hb content was measured in the three different side-streams with different treatments at the initial time point of the storage experiment (Day 0) using the acetone-based method originally developed by Hornsey (1956) [42] as modified in our recent study Wu et al. 2020 [13]. Around 50 g cod side-streams from the different treatments and fractions were carefully placed one by one in a porcelain mortar containing liquid nitrogen. Additional liquid nitrogen was slowly added, alternating sub-portions of the 50 g mince as needed to completely freeze the mince. The frozen mince was pounded using a pestle to break it into smaller pieces, which were then transferred to a 1 L stainless steel container for grinding into a fine powder. Milliliter amounts of liquid nitrogen were added as needed to keep the mince frozen and to facilitate grinding as well as further handling. Then, 4 g of side-stream powder was subsequently used to measure total heme. The difference between the absorbance values at 640 and 700 nm was used to calculate the heme concentration. Bovine Hb (0.1, 0.5, 1, 5, and 10 μM) was used to prepare a standard curve, and results were then expressed as μmol Hb/kg oil.

### 4.12. Crude Protein Content

For determination of crude protein content, the samples were combusted at 800 °C in an oxygen-rich atmosphere using DUMAS (Rapid MAX N exceed cube N/Protein analyser). Approx. 200 mg of side-stream were weighted (duplicate measurements). A conversion factor of 5.58 for fish materials [43] was used to determine the protein concentration in the samples.

### 4.13. Sensory Evaluation

The sensory evaluation was performed by six tested and trained assessors from the sensory panel at DTU Food. The panel was specifically trained in objective descriptive analysis, and the sensory evaluations were performed in a sensory lab with separated booths under normal daylight and at ambient temperature according to the standards and guidelines for the design and construction of sensory lab to ISO 5496, 2006; ISO 8586, 2012; ISO 8589, 2007; NMKL Procedure No. 6, 2023 [44,45,46,47].

The first sessions both for the evaluation of cold and frozen stored samples were used to develop a vocabulary to describe the sensory characteristics of the samples. Furthermore, the panel was trained using a scale describing the intensity of the attributes ensuring that all experimental conditions were represented. The scale was an unstructured 15 cm line scale with anchor point at 1.5 cm and 13.5 cm. All samples were served in random order with a three digital number, and the assessors were served peeled cucumber and water to clean their mouths between samples. It is only results for the sensory attributes that had developed or changed pronounced during storage that are shown.

The final vocabulary for the cold-stored samples was: Sweet; Fish meat (white fish); Green/plant stem/hay; Metallic/liver; Rancid; Off-odor.

The final vocabulary for the frozen-stored samples was: Sweet; Fish meat (white fish); Green/plant stem/hay; Metallic/liver; Rancid; Fishy, Carboard, Fatty.

### 4.14. Statistics

Results for the different analyses are reported as average ± standard deviation (n = 2). The program Statgraphic (Version 18.1.06, Statpoint Technologies, Inc., Warrenton, VA, USA) was employed for the statistical analysis. Mean value, standard deviation, and number of replicates were used for the analysis of variance (ANOVA). Multiple--sample comparison was performed to determine the statistical differences between sample treatments for the same side-stream and sampling points within same treatment using Tukey’s post hoc test. A significant level of α = 0.05 was applied.

## 5. Conclusions

Antioxidant treatment of the cod side-streams could effectively preserve the lipids against oxidation. In addition, no rancidity was detected by a trained sensory panel in the ice stored side-streams subjected to antioxidant dipping. An exception was the viscera samples stored frozen, for which only the treatment with high concentration of RE (Rosemary extract) inhibited development of rancidity. However, the development of PV and TBARS was lower when DM (Duralox MANC) was applied than with RE.

DM was the most effective antioxidant, followed by the lipophilic RE in a high concentration (0.2%). At frozen storage, DM inhibited the development of both PV and TBARS for nearly 3 months. Under ice storage, this antioxidant completely inhibited development of PV in backbone and viscera, and inhibited PV formation in heads for 4 days. Regarding TBARS, this oxidation marker was either fully inhibited (backbone), inhibited for 4 days (viscera), or decreased during storage and had the lowest level of all treatments (head). The present results facilitate a higher utilization degree of cod side-streams for production of new food or higher value ingredients since the lipids are kept stable during storage. Such extended storage is crucial for side-streams generated far away from the upcycling production site.

## Figures and Tables

**Table 1:** Composition including dry matter, protein content, oil content, percent eicosapentaenoic acid (EPA), and percent docosahexaenoic acid (DHA) of total fatty acids measured for the three different cod solid side-streams: backbone, head, and viscera. ^1^

Compositional Parameter	Backbone	Head	Viscera
Dry matter (%)	23.2 ± 1.8 ^a^	23.4 ± 1.6 ^a^	34.9 ± 5.3 ^b^
Oil (% ww)	1.2 ± 0.1 ^a^	5.6 ± 0.5 ^a^	22.5 ± 5.1 ^b^
EPA (% of total fatty acids)	15.2 ± 1.2 ^b^	10.5 ± 1.4 ^a^	9.4 ± 0.9 ^a^
DHA (% of total fatty acids	20.8 ± 0.7 ^b^	11.0 ± 1.2 ^a^	11.9 ± 0.8 ^a^
Protein (% ww)	15.3 ± 0.7 ^c^	13.6 ± 0.6 ^b^	10.3 ± 0.7 ^a^

^1^ The results are shown as average and STD with *n* = 6 (backbone and head). The viscera were packed in bags of 1 kg, and two bags were used for the characterization. Each bag was divided into three fractions before being fully thawed without taking into account whether or not all organs were represented in each fraction, i.e., a total of 6 fractions were used for the viscera. Different superscript letters for each compositional parameter indicate significant differences between the side-streams. Abbreviation: ww, wet weight.

**Table 2:** Experimental design including the different cod side-streams—backbone (B), head (H), and viscera (V)—treatments, and sample codes.

Treatments	Codes	Backbone (B)	Head (H)	Viscera (V)
No dipping	NoD	B_NoD	H_NoD	V_NoD
0.9% NaCl	Con	B_Con	H_Con	V_Con
0.9% NaCl and 2% Duralox MANC-213	DM_H	B_DM_H	H_DM_H	V_DM_H
0.9% NaCl and 0.05% rosemary extract	RE_L	B_RE_L	H_RE_L	V_RE_L
0.9% NaCl and 0.2% rosemary extract	RE_H	B_RE_H	H_RE_H	V_RE_H

## Data Availability

The data presented in this study are available in the article and as Supplementary Material.

## Supplementary Materials

The following are available online at https://www.mdpi.com/article/10.3390/md21110591/s1, Figure S1: Antioxidant-treated cod frame stored at −20 °C for 6 months, Figure S2: Antioxidant-treated cod head stored at −20 °C for 6 months, Figure S3: Antioxidant-treated mixed cod guts stored at −20 °C for 6 months. Table S1. Composition including dry matter, oil and protein content for the three different cod side-stream fractions, backbone, head and viscera. The content is reported as a range from the storage experiment of the different antioxidant treated fractions.
