# Supplementary material for: Oxidative Stability of Side-Streams from Cod Filleting—Effect of Antioxidant Dipping and Low-Temperature Storage
Source: Mar Drugs. 2023 Nov 13;21(11):591. doi: 10.3390/md21110591 (PMC10671878; doi:10.3390/md21110591)
Supplement: Supplementary file 1 [file marinedrugs-21-00591-s001.zip › marinedrugs-2666920-supplementary.pdf]

Supplementary material (S)

Table S1. Composition including dry matter, oil and protein content for the three different cod side-stream fractions, backbone, head and viscera. The content is reported as a range from the storage experiment of the different antioxidant treated fractions.

Compositional parameter	Backbone	Head	Viscera
Dry matter [%]	18.3 – 22.9	19.1 – 23.2	27.5 – 35.9
Oil [% ww]	1.0 – 1.3	1.3 – 2.8	12.7 – 21.0
Protein [% ww]	12.0 – 15.5	10.8 – 15.0	8.2 – 11.3

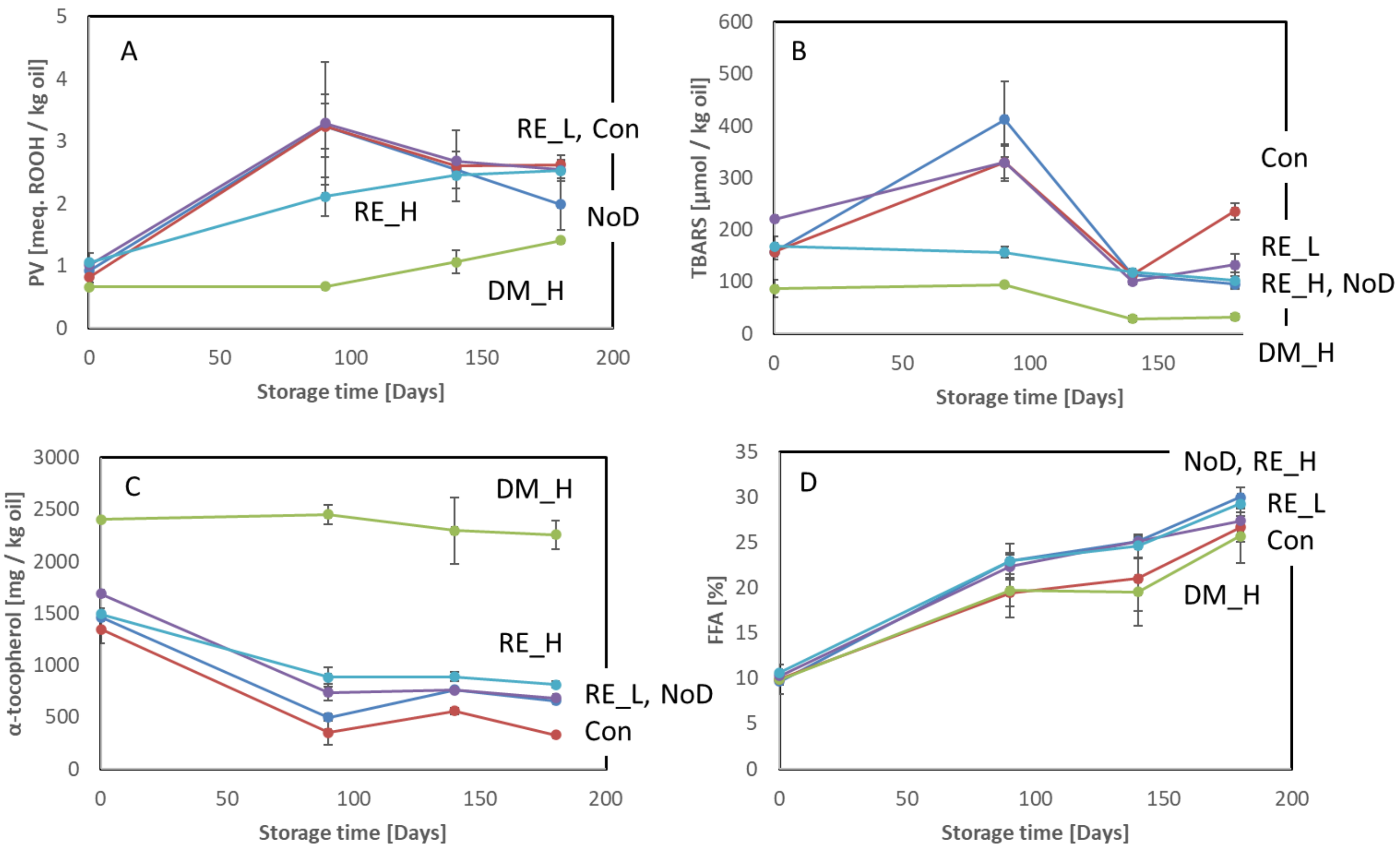

Figure S1. Antioxidant-treated and non-treated cod backbones stored at -20°C for 6 months A) PV, B) TBARS, C) α-tocopherol and D) FFA. NoD: No dipping; Con: Dipped in 0.9% NaCl solution; DM_H: Dipped in 2% Duralox MANC dissolved in 0.9% NaCl solution; RE_L: Dipped in 0.05% Rosemary extract dissolved in 0.9% NaCl solution; RE_H: Dipped in 0.2% Rosemary extract dissolved in 0.9% NaCl solution. The results which are expressed on a lipid basis are shown as average ± standard deviation (n=2).

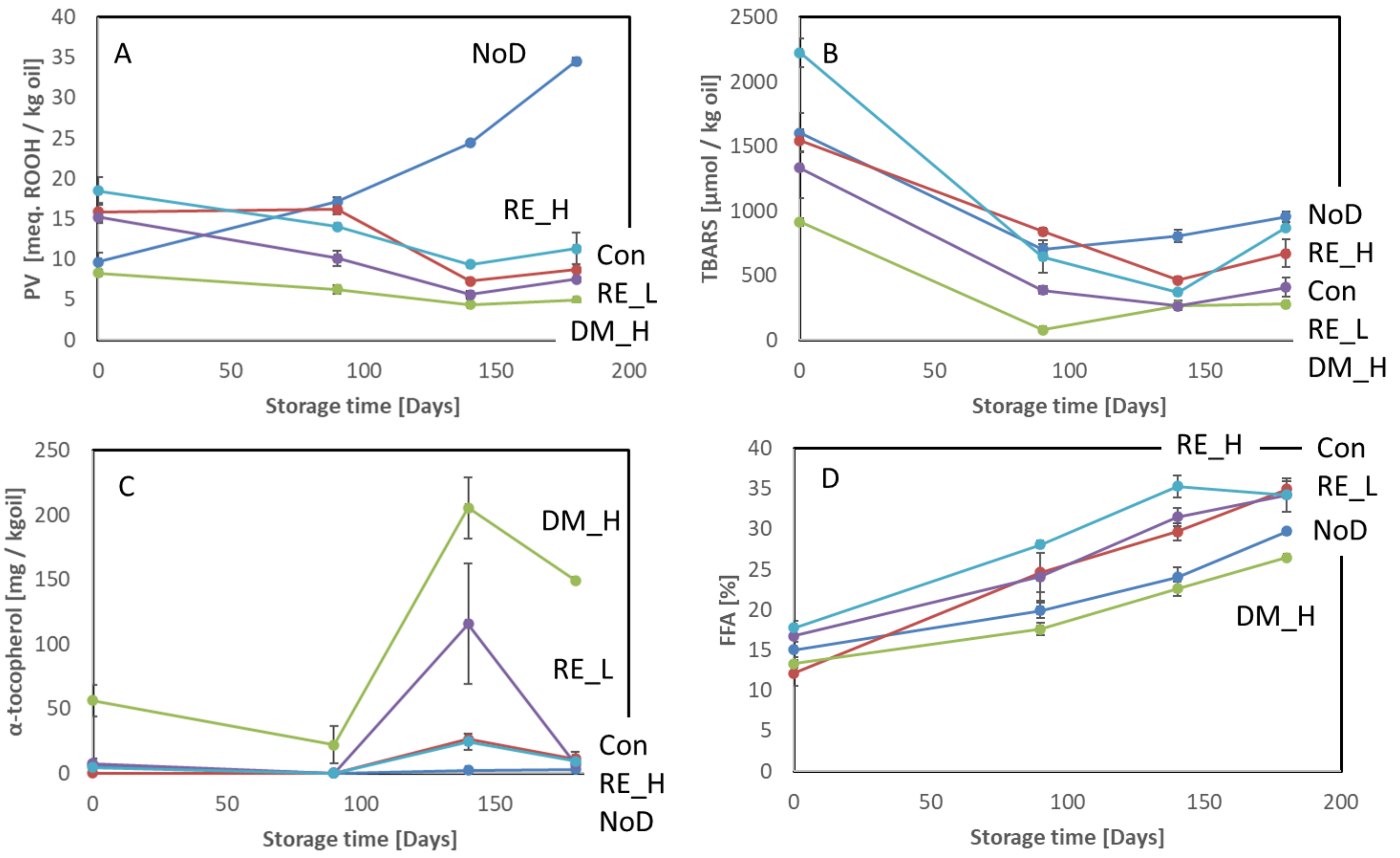

Figure S2. Antioxidant-treated and non-treated cod heads stored at -20°C for 6 months A) PV, B) TBARS, C) α-tocopherol and D)FFA. NoD: No dipping; Con: Dipped in 0.9% NaCl solution; DM_H: Dipped in 2% Duralox MANC dissolved in 0.9% NaCl solution; RE_L: Dipped in 0.05% Rosemary extract dissolved in 0.9% NaCl solution; RE_H: Dipped in 0.2% Rosemary extract dissolved in 0.9% NaCl solution. The results which are expressed on a lipid basis are shown as average ± standard deviation (n=2).

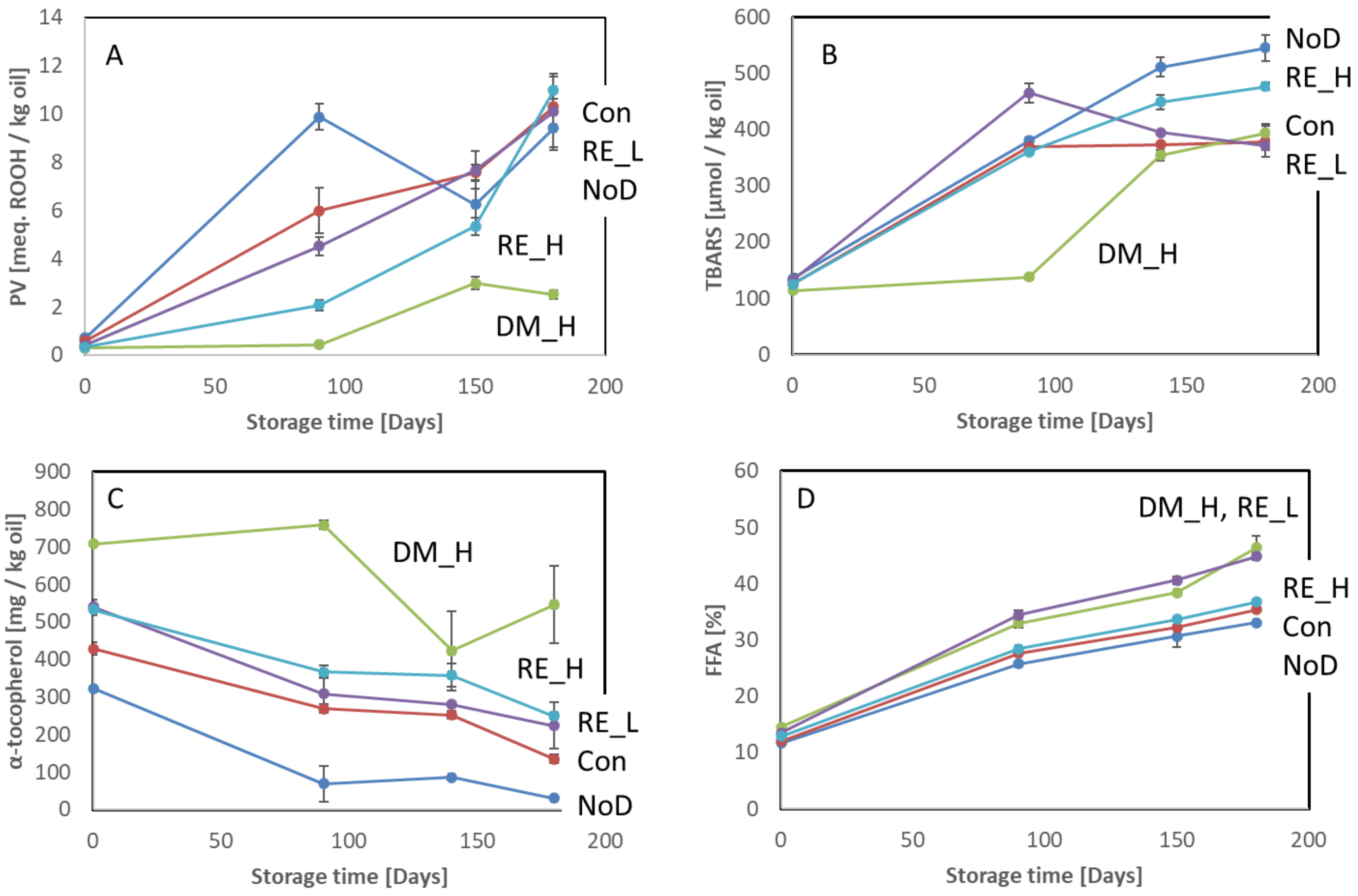

Figure S3. Antioxidant-treated and non-treated cod viscera fractions stored at -20°C for 6 months A) PV, B) TBARS, C) α-tocopherol and D) FFA. NoD: No dipping; Con: Dipped in 0.9% NaCl solution; DM_H: Dipped in 2% Duralox MANC dissolved in 0.9% NaCl solution; RE_L: Dipped in 0.05% Rosemary extract dissolved in 0.9% NaCl solution; RE_H: Diped in 0.2% Rosemary extract dissolved in 0.9% NaCl solution. The results which are expressed on a lipid basis are shown as average ± standard deviation (n=2).

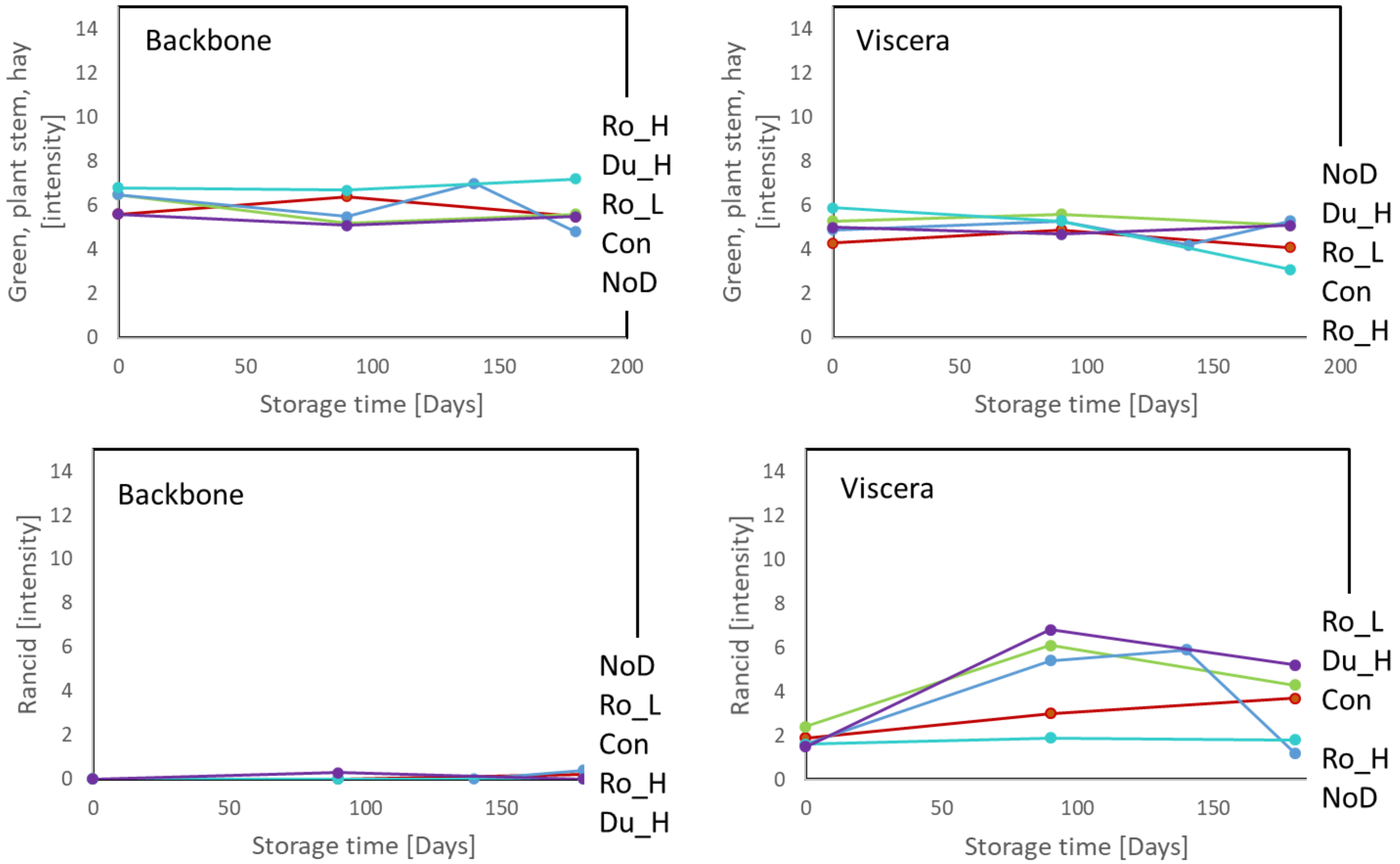

Figure S4. Sensory intensity of green/plant stem/hay and rancid for antioxidant-treated and non-treated cod backbones and viscera fractions stored at -20°C for 6 months. NoD: No dipping; Con: Dipped in 0.9% NaCl solution; DM_H: Dipped in 2% Duralox MANC dissolved in 0.9% NaCl solution; RE_L: Dipped in 0.05% Rosemary extract dissolved in 0.9% NaCl solution; RE_H: Dipped in 0.2% Rosemary extract dissolved in 0.9% NaCl solution.
